# Analysis of risk factors for postoperative delirium in middle-aged and elderly fracture patients in the perioperative period

**DOI:** 10.1038/s41598-023-40090-z

**Published:** 2023-08-10

**Authors:** Zhongcheng An, Liangen Xiao, Chen Chen, Lianguo Wu, Hao Wei, Xiaoping Zhang, Liqiang Dong

**Affiliations:** 1 Department of Orthopedics, The Second Affiliated Hospital of Zhejiang Chinese Medical University, Hangzhou, 310005 Zhejiang People’s Republic of China; 2 The Second Clinical Medical College of Zhejiang Chinese Medical University, Hangzhou, 310005 Zhejiang People’s Republic of China

**Keywords:** Medical research, Risk factors

## Abstract

To investigate the incidence rate and risk factors of postoperative delirium in middle-aged and elderly patients with fracture. A total of 648 middle-aged and elderly fracture patients who underwent surgical treatment in our hospital from January 2018 to December 2020 were included in the study, aged 50–103 years, mean 70.10 ± 11.37 years. The incidence of postoperative delirium was analyzed. Univariate analysis was used to screen the risk factors of gender, age, interval between injury and operation, preoperative complications, fracture site, anesthesia method, operation time, intraoperative blood loss, hidden blood loss and hormone use. For the factors with *P <* 0.05, multivariate logistic regression analysis was used to determine the main independent risk factors. 115 cases (17.74%) of 648 patients had postoperative delirium. Univariate analysis showed that patients with delirium and patients without delirium had significant correlation in age, medical disease comorbidity, fracture type, anesthesia method, operation time and perioperative blood loss (*P <* 0.05). Multivariate logistic regression analysis showed that age (OR = 1.061), preoperative complications (OR = 1.667), perioperative blood loss (OR = 1.002) were positively correlated with postoperative delirium. It shows that older age, more preoperative complications, longer operation time and more perioperative bleeding are more likely to lead to postoperative delirium; patients with general anesthesia were more likely to develop postoperative delirium than patients with local anesthesia (OR = 1.628); and patients with hip and pelvic fractures are more likely to develop a postoperative delirious state (OR = 1.316). Advanced age, complex orthopedic surgery, more medical comorbidities, general anesthesia and greater perioperative blood loss may be independent risk factors for the development of delirium after internal fixation of fractures in middle-aged and elderly patients.

## Introduction

With the aging of the world population, the number of middle-aged and elderly orthopedic patients is increasing year by year, and postoperative delirium (POD) is becoming more and more common as a postoperative complication in middle-aged and elderly fracture patients. POD usually occurs 2–3 days after operation, and the incidence of POD in patients over 65 years old is 5–50%. It will not only lead to prolonged hospital stay, increased nursing costs, but also lead to permanent dementia, even life-threatening ^1–3^, and become an important focus of orthopedic physicians. Currently, there are still difficulties in the diagnosis and treatment of POD, especially the lack of effective therapeutic drugs. Therefore, it is urgent to prevent the incidence of POD and shorten the duration of delirium by targeting high-risk patients at an early stage.

Given that the pathophysiological mechanism of POD is still unclear, and there are multiple subtypes in clinical practice, the risk factors leading to its occurrence have not been clarified so far. Therefore, it is important to clarify the mechanism and risk factors of POD and to formulate reasonable prevention and treatment measures to reduce the incidence of POD in middle-aged and elderly fracture patients. In this paper, we retrospectively analyzed the clinical data of 648 middle-aged and elderly (≥ 50 years old) fracture patients who underwent surgical treatment in our hospital between January 2018 and December 2020, counted the incidence of POD, discussed and analyzed the risk factors related to POD in middle-aged and elderly patients with fractures, and provided a basis for the prevention and treatment of POD.

## Materials and methods

### Inclusion and exclusion criteria

Inclusion criteria: ① age ≥ 50 years old; ② no previous family history of mental illness, no history of mental disorder, no cognitive impairment; ③ clinical symptoms and imaging data were diagnosed as fracture; ④ surgical treatment was performed without contraindication of anesthesia operation; ⑤ no use of drugs that can significantly affect mental activity in the last 1 month; ⑥ no history of heart, brain or lung surgery, no history of paralysis or convulsions.

Exclusion criteria: ① preoperative patients with a clear history of neurological and psychiatric disorders and taking corresponding medications; ② preoperative patients with dementia with cognitive impairment; ③ patients with severe visual or hearing diseases cannot cooperate with the completion of cognitive function tests; ④ patients with conservative treatment of fractures.

### General information

A total of 648 patients were included in this study, male 256 cases and female 392 cases; age ranged from 50 to 103 years, mean 70.10 ± 11.37 years; cases of extremity fractures 239, cases of spine fractures 212, and 197 cases of hip and pelvis fractures. 330 cases were combined with medical diseases. The time from injury to surgery ranged from 4 to 72 h, mean 37.35 ± 19.81 h.

### Diagnostic criteria for postoperative delirium^4^

The main diagnosis of POD is based on the American Psychiatric Association's Mental Disorders and Statistics Manual (5th Edition) (DSM-5) and the Ambiguous Consciousness Assessment Method (CAM). The main diagnostic features include acute or subacute attacks, repeated or fluctuating conditions, clinical manifestations of inattention, change of consciousness and decrease of cognitive function (orientation disorder, memory disorder, language disorder). Other features that support the diagnosis of POD include sleep–wake cycle disorders, perceived disorders (hallucinations or misunderstandings, etc.), delusions, improper behavior, emotional instability, etc.

### Observation index

Refer to relevant literature research and clinical experience to determine the relevant factors that may affect the occurrence of delirium after orthopedic surgery in middle-aged and elderly patients. Record the patient’s gender, age, interval between injury and operation, preoperative complications, fracture site, anesthesia method, operation time, intraoperative blood loss, hidden blood loss and hormone use, etc.

### Statistical methods

SPSS 23.0 statistical software was used for statistical analysis of the data. Measurement data were expressed as mean ± standard deviation, t-test was used for comparison between two groups, and chi-square test was used for count data comparison between two groups. *P <* 0.05 was considered statistically significant. Finally, all the observed indicators were taken as univariate covariates, and postoperative delirium was taken as the dependent variable, which was taken into the Logistic regression model for multivariate analysis. The 95% confidence interval (CI) was used, and *P <* 0.05 was considered statistically significant.

### Ethical approval and consent to participate

The study was approved by the Institutional Review Board (IRB) of Second Affiliated Hospital of Zhejiang Chinese Medical University (authorization number 2020-KL-040-01), all methods were carried out in accordance with relevant guidelines and regulations. This study was carried out in compliance with the Declaration of Helsinki. Due to the retrospective nature of the study, Second Affiliated Hospital of Zhejiang Chinese Medical University’s IRB waived the need of obtaining informed consent.

## Results

### Incidence of postoperative delirium

A total 648 of patients met the inclusion and exclusion criteria for inclusion in this study. Postoperative 115 cases of middle-aged and elderly fracture patients developed POD with an overall incidence of 17.74%, including cases of extremity fractures 38, spine fractures 28, and hip and pelvis fractures 49.

### Univariate analysis

Univariate analysis showed that the incidence of postoperative delirium in middle-aged and elderly patients with fracture was correlated with age, preoperative complications, fracture type, anesthesia method, operation time and perioperative blood loss, and the difference was statistically significant (*P <* 0.05). There was no significant correlation with the patient's gender, the interval from injury to surgery, and hormone use, and the difference was not statistically significant (*P* > 0.05). The specific analysis results are shown in Table 1.

**Table 1:** Univariate analysis of postoperative delirium in middle-aged and elderly patients with fracture.

Observation index	Delirium group	Delirium-free group	T/x^2^ value	*P* value
Gender			0.008	0.928
Male	45	211		
Female	70	322		
Age	76.55 ± 12.60	68.73 ± 10.60	-6.197	0.000*
Interval between injury and operation (h)	35.23 ± 19.5	37.81 ± 19.87	1.264	0.207
Preoperative complications			4.869	0.027*
Yes	70	264		
No	45	269		
Fracture type			10.405	0.006*
Extremity fractures	38	201		
Spinal fracture	28	184		
Hip and pelvis fractures	49	148		
Anesthesia method			6.437	0.011*
Local anesthesia	54	319		
General anesthesia	61	214		
Operation time (h)	2.35 ± 1.12	2.12 ± 1.08	-2.035	0.042*
Perioperative blood loss (ml)	313.27 ± 158.54	263.06 ± 156.93	-3.106	0.002*
Hormone use			3.325	0.068
Yes	76	303		
No	39	230		

### Logistic regression analysis of related factors

The indicators that showed statistical differences in the univariate analysis results were included in the logistic regression model to test the reality. Among them, age, preoperative complications, operation time, perioperative blood loss were positively correlated with the occurrence of POD. This means that the older the age, the more preoperative complications, the longer the operation time, the more perioperative blood loss, the more likely to cause POD. Patients with general anesthesia are also more prone to POD than patients with local anesthesia. The specific analysis results are shown in Table 2.

**Table 2:** Logistic regression analysis of factors related to postoperative delirium in middle-aged and elderly patients with fractures.

Related factors	B value	SE value	Wald value	*P* value	OR value	95%CI
Age	0.059	0.010	37.625	0.000*	1.061	1.041 ~ 1.081
Preoperative Complications	0.511	0.223	5.266	0.022*	1.667	1.077 ~ 2.579
Fracture type	0.275	0.133	4.263	0.039*	1.316	1.014 ~ 1.708
Anesthesia method	0.487	0.219	4.936	0.026*	1.628	1.059 ~ 2.501
Operation time (h)	0.121	0.100	1.474	0.225	1.129	0.928 ~ 1.372
Perioperative blood loss (ml)	0.002	0.001	7.688	0.006*	1.002	1.001 ~ 1.003

## Discussion

POD is a common surgical complication, especially in middle-aged and elderly surgical patients^5^, which is harmful to patients and families and affects their prognosis and recovery. The clinical symptoms of postoperative delirium are often difficult to recognize and easy to ignore, requiring keen clinical observation skills. The pathogenesis of postoperative delirium in middle-aged and elderly patients with fractures is still unclear, although some scholars have proposed some theoretical hypotheses, including the inflammatory theory, the neurotransmitter theory, and the theory of altered brain function, which may contribute to the occurrence of postoperative delirium^6^. However, there is a lack of clinical studies to support them.

### Relationship between postoperative delirium and age

At present, most scholars at home and abroad generally believe that advanced age is an independent risk factor for postoperative delirium in middle-aged and elderly patients, especially in patients with hip surgery^7,8^. Some scholars believe that this may be related to the decline of cholinergic nerves in the brain, the gradual decline of acetylcholine and central cholinergic neurons with age, systemic stress and neurotransmitter disorders, as well as the decline of cortical function, resulting in abnormal brain function^9^. At the same time, as the body functions deteriorate, the ability of middle-aged and elderly patients to adapt to external stress decreases, especially for fracture trauma and surgical stimulation, which leads to a decrease in their ability to adapt to the surrounding environment, and they are more likely to be affected by psychological factors before surgery, which leads to nervousness, apprehension and fear, and makes them overly nervous, which, together with trauma or surgical stress, increases the risk of delirium. This study shows that the risk of postoperative delirium in patients aged ≥ 70 years old is much higher than that in patients aged < 70 years old.

### Relationship between postoperative delirium and complications

Middle-aged and elderly patients are often complicated with a variety of medical diseases, such as "three highs"(high blood pressure, high blood sugar, high blood lipid), heart disease, lung disease, etc. This can easily lead to decreased stress ability and immunity of the body, water and electrolyte disorders, acid–base imbalance, and postoperative hypoxemia, leading to brain cell edema and brain dysfunction, which leads to POD. Previous studies have found that some preoperative underlying diseases such as hypertension and coronary heart disease are related to postoperative delirium, and the acute onset of chronic diseases is the most common precipitating factor. The more complications there are, the more precipitating factors there are, especially for patients with preoperative Alzheimer's disease and pulmonary diseases, the incidence of POD is higher^10^. Also, patients with more complicated internal diseases are more likely to suffer from electrolyte disorder and POD after surgery^11^. Related studies have confirmed that higher American Society of Anesthesiologists (ASA) scores is a high risk factor for postoperative delirium^12^, which explains why POD often occurs in elderly and weak adults, but rarely in young people.

### Association of postoperative delirium with type of surgery and length of bed rest

Current studies have found that the incidence of POD in middle-aged and elderly patients after hip and spine surgery is significantly higher than that in other patients with simple extremity fractures^13,14^. A recent meta-analysis showed that hip surgery was significantly associated with the occurrence of POD in elderly patients after orthopedic surgery^15^. This may be related to the fact that patients still need to rest in bed for a long time after the operation of these parts. Meanwhile, a prospective study also confirmed that the occurrence of POD is closely related to the decrease of postoperative activity^16^. Zhu et al.^17^ also confirmed through a meta-analysis that POD after spinal surgery is associated with a significant reduction in early postoperative activities. Therefore, the above results can confirm that the incidence of POD in patients is significantly increased after major surgeries such as spine and hip surgery, and at the same time, a long time of bed rest after surgery is also easy to lead to the occurrence of POD in patients. This is similar to the results of our study.

### Relationship between postoperative delirium and anesthetic methods

The pathophysiological mechanism of POD induced by anesthesia remains unclear. Previous studies have confirmed that it may be related to the dose of anesthetic drugs used during the operation, hemodynamic impairment and inflammatory response. At present, many scholars have explored the correlation between anesthesia methods and POD. Weber et al.^18^ showed that the cognitive function of patients after general anesthesia decreased more significantly than that after local anesthesia. Vasilevskis et al.^19^ showed that POD was highly likely to occur in middle-aged and elderly patients after general anesthesia. Some scholars have proved that local anesthesia can significantly reduce the occurrence of POD in the early postoperative period^20^. This may explain why POD is less common in patients with osteoporotic vertebral compression fractures after percutaneous vertebral augmentation. In this way, the occurrence of POD in early postoperative period also has a certain relationship with anesthesia methods. This study showed that the incidence of POD in patients with general anesthesia was significantly higher than that in patients with local anesthesia. Therefore, for middle-aged and elderly patients with fracture, we should mainly pay attention to the management of surgery and anesthesia. For the middle-aged and elderly patients, surgical methods with less trauma and short time should be selected as much as possible, and the normal physiological functions of the human body should not be disturbed as much as possible. At the same time, the anesthesia method should be as simple as possible, if necessary, electroencephalogram monitoring can be used in the operation to avoid too deep anesthesia. However, a recent multicenter randomized controlled study confirmed that there was no significant difference in the incidence, type and severity of postoperative delirium between local and general anesthesia in elderly patients with hip fracture^21^. Neuman m.d. et al.^22^ found through clinical research that the incidence of postoperative delirium in elderly patients with hip fractures under spinal anesthesia and general anesthesia was similar. These studies changed the traditional concept that "general anesthesia can cause brain dysfunction in elderly patients" and provided important guidance for clinical practice.

### Relationship between postoperative delirium and hormone use

Glucocorticoids have anti-allergic, anti-inflammatory and other effects. Perioperative use of glucocorticoids in spinal surgery can reduce the stress response of patients and play a certain neuroprotective role. Therefore, after spinal surgery with neurological dysfunction, most doctors prefer to give patients a certain amount of glucocorticoids in the short term. A meta-analysis found that reducing the use of methylprednisolone in middle-aged and elderly patients after spinal surgery can reduce the incidence of POD^23^. Clemmesen et al.^24^ conducted a randomized controlled study on 117 elderly patients with hip fracture, found that preoperative intravenous injection of 125 mg methylprednisolone could significantly reduce the incidence of postoperative delirium, but did not reduce the severity of postoperative delirium. However, Royse et al.^25^ found that methylprednisolone could not reduce the incidence of POD through a randomized double-blind trial. At the same time, Sauër et al.^26^ found that the use of dexamethasone similarly failed to reduce the occurrence of POD. In the present study, we found that in some patients with burst spine fractures and hip fractures, the short-term use of methylprednisolone in small amounts (≤ 48 h) during the perioperative period had no significant effect on the occurrence of POD. Therefore, it is controversial whether there is an effect of hormonal use on POD occurrence.

### Relationship between postoperative delirium and perioperative bleeding

At present, most studies have confirmed that intraoperative bleeding and postoperative hidden blood loss can significantly increase the incidence of POD. A recent meta-analysis showed that more blood loss during spinal surgery can be used to predict the occurrence of POD^17^. Nazemi et al.^22^ also confirmed that more blood loss during spinal surgery is more likely to lead to the occurrence of POD. Li et al.^11^ confirmed that high hidden blood loss during the perioperative period was an independent risk factor for the development of POD after internal fixation in middle-aged and elderly patients with intertrochanteric femoral fractures. This study showed that the incidence of POD in the operation with high perioperative blood loss such as spine and hip surgery was significantly higher than that in the operation with simple extremity fractures which could use tourniquet. This may be due to the increase of perioperative blood loss, leading to postoperative hypotension, interfering with the stability of blood circulation, affecting blood pressure and tissue perfusion and oxygen supply, contributing to tissue ischemia and hypoxia, and inducing POD.

## Limitations

This study was a retrospective analysis, and the inclusion and exclusion criteria for patients with POD may be biased. In addition, this study was reported from a single medical clinical center, and the mechanisms and influencing factors regarding the occurrence of delirium after internal fixation of fractures in the middle-aged and elderly still need to be further explored in a multicenter, large sample size study.

## Conclusions

In summary, advanced age, complex orthopedic surgery, more medical comorbidities, general anesthesia, greater perioperative blood loss may be independent risk factors for the development of POD after internal fixation of fractures in middle-aged and elderly patients. In middle-aged and elderly patients with fractures, less surgical trauma and more comprehensive perioperative management with guaranteed efficacy may significantly reduce the incidence of postoperative delirium.

## Data Availability

The datasets generated and analyzed during the current study are available from the corresponding author on reasonable request.

## Abbreviations

POD: Postoperative delirium
DSM-5: American Psychiatric association's mental disorders and statistics manual
CAM: Ambiguous consciousness assessment method
CI: Confidence interval
ASA: American society of anesthesiologists
